# Antimicrobial Resistance Genotypes and Mobile Genetic Elements of Poultry-Derived *Escherichia coli*: A Retrospective Genomic Study from the United States

**DOI:** 10.3390/pathogens14080726

**Published:** 2025-07-23

**Authors:** Sohyun Cho, Hazem Ramadan, Lari M. Hiott, Jonathan G. Frye, Charlene R. Jackson

**Affiliations:** 1 Egg and Poultry Production Safety Research Unit, United States National Poultry Research Center (USNPRC), Agricultural Research Service (ARS), United States Department of Agriculture (USDA), Athens, GA 30605, USA; sohyun.cho@usda.gov; 2 Hygiene and Zoonoses Department, Faculty of Veterinary Medicine, Mansoura University, Mansoura 35516, Egypt; hazem_hassan@mans.edu.eg; 3 Poultry Microbiological Safety and Processing Research Unit, United States National Poultry Research Center (USNPRC), Agricultural Research Service (ARS), United States Department of Agriculture (USDA), Athens, GA 30605, USA; lari.hiott@usda.gov (L.M.H.); jonathan.frye@usda.gov (J.G.F.)

**Keywords:** *E. coli*, poultry, antibiotic resistance

## Abstract

The presence of antibiotic resistance in commensal bacteria may be an influential factor in the persistence of resistance in pathogens. This is especially critical for *Escherichia coli* that consumers may be exposed to through the consumption of uncooked meat. In this study, *E. coli* isolates previously recovered from poultry in the US between 2001 and 2012 were whole-genome sequenced to identify their antibiotic resistance genes and mobile genetic elements. The genomes of 98 *E. coli* isolates from poultry carcass rinsates and 2 isolates from poultry diagnostic samples with multidrug resistance or potential extended-spectrum β-lactam (ESBL)-producing phenotypes as well as the genetic variabilities among the *E. coli* were assessed. All *E. coli* isolates were positive for at least one antibiotic resistance gene and plasmid replicon, with 37 resistance genes and 27 plasmid replicons detected among the isolates. While no ESBL genes were detected, *bla*_CMY-2_ was the most common β-lactamase gene, and *bla*_TEM_ and *bla*_CARB-2_ were also identified. Most isolates (95%) harbored at least one intact phage, and as many as seven intact phages were identified in one isolate. These results show the occurrence of antibiotic resistance genes and mobile genetic elements in these 100 poultry-associated *E. coli* isolates, which may be responsible for the resistance phenotypes exhibited by the isolates. This retrospective study also enables comparisons of resistance genes and mobile genetic elements from more recent *E. coli* isolates associated with poultry to aid in understanding the trends of both antibiotic resistance phenotypes and genotypes in the poultry setting over time.

## 1. Introduction

The bacterial contamination of food products from poultry continues to be one of the leading contributors of foodborne illness [1,2]. These illnesses can be more difficult to treat if the bacteria are resistant to antibiotics as antibiotic resistance reduces the effectiveness of the drugs as a therapeutic option. Antibiotics were used in previous years in poultry production to not only treat, prevent, or control disease but also for growth promotion. However, the use of antibiotics of human clinical importance in food animal production is restricted due to changes in the antibiotics approved or available for use in food animals; also, consumer demand for “No Antibiotic Ever” products is growing [3,4]. Limiting the widespread use of antibiotics in the food animal sector lessens overall antibiotic selective pressure, which is expected to decrease the prevalence of both resistant foodborne bacteria and resistant commensal bacteria.

*Escherichia coli,* which are commonly described as commensals of the intestinal tract of mammals, can be found in a multitude of diverse environments, including water and soil [5,6]. Resistance to antibiotics, such as the aminoglycosides, β-lactams, and fluoroquinolones used to treat Gram-negative bacterial infections, has been commonly described in *E. coli* [7]. Carbapenem-resistant Enterobacteriaceae (CRE) and extended-spectrum β-lactam (ESBL)-producing Enterobacteriaceae, including *E. coli*, have maintained their status as urgent and serious antibiotic resistance threats to human health since 2013 as these bacteria are resistant to a number of β-lactam antibiotics, including penicillin, aztreonam and first-, second-, and third-generation cephalosporins [8,9]. They can also contribute to resistance to other antibiotic classes, such as aminoglycosides, fluoroquinolones, sulfonamides, and tetracyclines, as ESBL-encoding plasmids may also harbor resistance genes to those drugs [10,11,12].

Plasmids and other mobile genetic elements are common in commensal *E. coli,* enabling these bacteria to acquire and disseminate antibiotic resistance genes among other bacteria. Antibiotic resistance and the resistance mechanisms in *E. coli* are similar to those found in *Salmonella*, and studies have shown that plasmids and antibiotic resistance genes can be transferred between *E. coli* and *Salmonella* [13,14], suggesting that *E. coli* could serve as a reservoir and donor of resistance to *Salmonella* and other foodborne pathogens. Drug-resistant non-typhoidal *Salmonella* is a concern, especially those that are resistant to azithromycin, ceftriaxone, and ciprofloxacin, which are antibiotics used to treat the most severe cases of salmonellosis [8].

There are many challenges when attempting to determine strategies to reduce human exposure to foodborne bacteria. The seeming ubiquity of sources of commensal *E. coli* can be attributed to a diverse and variable bacterial genome, resulting from DNA rearrangements, point mutations, horizontal gene transfer, and gene gain and loss [15]. However, despite these genetic events, commensal *E. coli* maintain a core genome that allows for the determination of phylogenetic relationships among isolates using core genome Multilocus Sequence Typing (cgMLST), hierarchical clustering (HierCC) of cgMLST, single-nucleotide polymorphisms (SNPs) and phylotypes from whole-genome sequence analysis.

Whole-genome sequencing is a technique that represents a major advancement in sequencing technology and enables the determination of a whole bacterial genome sequence in a matter of days. The National Antimicrobial Resistance Monitoring System (NARMS) was established by the Centers for Disease Control and Prevention (CDC), the US Food and Drug Administration (FDA), and the US Department of Agriculture (USDA) to monitor antibiotic resistance in foodborne and enteric bacteria from human and animal clinical specimens, from healthy farm animals, and from raw products of food-producing animals at slaughter. Historically, the characterization of bacterial isolates for the NARMS program included phenotypic and genotypic analysis, but recently, the NARMS program has adopted the use of whole-genome sequencing to replace several analytic methods for the characterization of antibiotic resistance. Thus, this retrospective study sequenced the whole genomes of antibiotic-resistant *E. coli* isolates that were recovered as part of NARMS before WGS was standard practice.

This study aimed to investigate diversity among antibiotic-resistant commensal *E. coli* from poultry and the genetic environment of resistance, including factors responsible for the spread and maintenance of multidrug resistance (MDR; resistance to three or more antibiotic classes). The genomes of previously tested MDR or potentially ESBL-producing (determined by ceftriaxone resistance) *E. coli* from these NARMS isolates were sequenced to investigate the diversity of antibiotic resistance phenotypes, antibiotic resistance genes, and mobile genetic elements, including plasmids and prophages, that can facilitate the transfer of antibiotic resistance genes through horizontal gene transfer.

## 2. Materials and Methods

Bacterial strains of *E. coli* in this study were selected from the USDA-Agricultural Research Service (ARS) bacterial isolate collection. The collection contains over 29,000 *E. coli* isolates from poultry, swine, cattle, and other non-food animal sources, collected between 2001 and 2012 as part of the USDA Food Safety and Inspection Service (FSIS) Hazard Analysis and Critical Control Points (HACCP) risk-based inspection program and other foodborne bacterial studies. All the isolates in the collection were previously tested for resistance to antibiotics, and selection of isolates for this study was based on MDR phenotype or potential ESBL production, determined by resistance to ceftriaxone. Microsoft Excel was utilized to randomly select 100 *E. coli* from healthy poultry carcass rinsates (*n* = 98) and poultry diagnostic samples (*n* = 2) from the collection database.

Antimicrobial susceptibility testing: Even though all the selected isolates were previously tested for resistance to antibiotics, their minimum inhibitory concentrations (MIC; μg/mL) were determined again for the same set of antibiotics as some antibiotics and breakpoints had changed over time. Broth microdilution was carried out according to the manufacturer’s directions using the Sensititre^TM^ semi-automated antimicrobial susceptibility system (Trek Diagnostic Systems, Cleveland, OH, USA) and the Sensititre^TM^ Gram-Negative Custom Plate CMV4AGNF. Antibiotics and breakpoints (in parenthesis) were as follows: amoxicillin/clavulanic acid (≥32/16 µg/mL), ampicillin (≥32 µg/mL), cefoxitin (≥32 µg/mL), ceftriaxone (≥4 µg/mL), chloramphenicol (≥32 µg/mL), ciprofloxacin (≥1 µg/mL), gentamicin (≥16 µg/mL), meropenem (≥4 µg/mL), nalidixic acid (≥32 µg/mL), sulfisoxazole (≥512 µg/mL), tetracycline (≥16 µg/mL), and trimethoprim/sulfamethoxazole (≥4/76 µg/mL). Results were interpreted according to Clinical and Laboratory Standards Institute (CLSI) guidelines when defined [16]. For azithromycin and streptomycin with no defined CLSI interpretive criteria, ≥32 µg/mL and ≥64 µg/mL were used, respectively, using interpretive criteria defined by the NARMS [17]. For the analysis, isolates identified as intermediate were considered susceptible to the drug. *E. coli* ATCC 25922, *Pseudomonas aeruginosa* ATCC 27853, *Enterococcus faecalis* ATCC 29212, and *Staphylococcus aureus* ATCC 29213 were used as controls for determination of MICs.

DNA extraction, genome sequencing, assembly and annotation: Genomic DNA from *E. coli* was extracted using the GenElute Bacterial Genomic DNA Kit (MilliporeSigma, Burlington, MA, USA). The qubit double-stranded DNA (dsDNA) high-sensitivity (HS) test kit (Invitrogen, Carlsbad, CA, USA) was used to quantify the extracted DNA in accordance with the manufacturer’s instructions (Life Technologies, Waltham, MA, USA). Illumina libraries were then prepared using the Nextera XT DNA library preparation kit and Nextera XT index set A primers (Illumina, San Diego, CA, USA). Using a 500-cycle MiSeq reagent kit version 2 (Illumina), paired-end sequences of 2 × 250 bp length were generated from DNA libraries on an Illumina MiSeq platform. The quality check of the generated short-read sequences was performed using FastQC (version 0.11.9), and sequences were then de novo assembled into contigs using A5-miseq assembler [18]. The assembled FASTA sequences were uploaded into Center for Genomic Epidemiology (CGE, https://www.genomicepidemiology.org/) and screened for the presence of antibiotic resistance genes and plasmid replicon types via ResFinder 4.1 [19] and PlasmidFinder 2.1 [20], respectively. Phages were identified using PHASTER [21], and only prophages with intact completeness were considered for report [22]. The sequence data were deposited into the National Center for Biotechnology Information (NCBI) under BioProject numbers PRJNA828150 and PRJNA1112887, and the accession numbers, along with the genome statistics, are listed in Supplementary Table S1.

Phylogenetic analysis: FASTQ files of the examined *E. coli* isolates were imported into Enterobase (https://enterobase.warwick.ac.uk/, version 1.2.0) and compared using single-nucleotide polymorphisms (SNPs) [23] and hierarchical clustering of cgMLST (HierCC) [24] based on 2513 core genomic loci. To analyze SNPs, isolates were mapped to the reference strain *E. coli* K-12 MG1655 and designated as HC200, differing by ≤200 core genomic alleles. Phylotyping of *E. coli* isolates was determined using version 0.6.2 of EzClermont [25].

Statistical analysis: To ascertain the relationship between resistance genes and plasmid replicons among the examined isolates, a correlation analysis was carried out. The resistance genotype and plasmid replicon findings were transformed into binary data (0/1); scores of 0 were assigned to absence of a resistance gene or a replicon type, while scores of 1 were assigned to the existence of resistance genes or replicon types. Binary data were imported into R (version 4.4.2), and using “corrplot” package’s functions “cor” and “cor.mtest”, correlations were calculated at a significance level of *p* < 0.05. Using the R packages “heatmap” (version 1.0.12) and “RColorBrewer” (version 1.1-3), a hierarchical heatmap was also created to group the isolates based on their antibiotic resistance genes, plasmid replicon types, and phylotypes.

## 3. Results and Discussion

### 3.1. Antibiotic Resistance Phenotypes of E. coli Isolates

Susceptibility testing results showed that 99 out of 100 *E. coli* isolates exhibited varying resistance to the 14 drugs tested. The most commonly observed resistance was against ampicillin (66/100; 66%) and tetracycline (62%). About half of the *E. coli* isolates were resistant to streptomycin (54%), amoxicillin/clavulanate, cefoxitin, ceftriaxone, gentamicin (49% each), nalidixic acid (46%), and ciprofloxacin and sulfisoxazole (42% each). This was followed by resistance to trimethoprim/sulfamethoxazole (19%), while resistance to azithromycin and chloramphenicol (7%) was least common. No resistance to meropenem was observed among the isolates tested.

One isolate was pan-susceptible to all the drugs tested, whereas MDR, defined as the resistance to at least three different classes of antibiotics, was observed in 63 isolates (Table 1). Ceftriaxone resistance, which is frequently used as a proxy for predicting the production of ESBLs [26,27], was observed in 49 isolates. One isolate was resistant to all the antibiotics tested except meropenem, exhibiting resistance to 13 drugs. *E. coli* isolates exhibited 39 antibiotic resistance phenotype patterns altogether, out of which resistance to amoxicillin/clavulanic acid, ampicillin, cefoxitin, and ceftriaxone (AugAmpFoxAxo) was most frequently observed (Table 1). This resistance pattern against the four antibiotic drugs was exhibited by about half the isolates (*n* = 49), with or without resistance to other drugs, indicating its widespread presence in *E. coli* from poultry carcass rinsates taken during 2001–2012. While this pattern shows resistance to only one antibiotic class, β-lactam, the next most common antibiotic resistance pattern, was an MDR pattern present in 12 isolates, exhibiting resistance to ciprofloxacin, gentamicin, nalidixic acid, and tetracycline.

Of the 100 isolates tested in the current study, 88 isolates were either MDR and/or resistant to ceftriaxone. All the isolates were from the 2001–2012 collection, and they were previously tested for resistance to antibiotics and determined to have either MDR or a potentially ESBL-producing phenotype. However, since they were tested against different sets of antibiotics and the breakpoints of the drugs changed over time, susceptibility testing was conducted on all the isolates again against the same set of antibiotics for consistency. This resulted in one isolate that was susceptible to all 14 drugs tested and 11 other isolates that were neither MDR nor resistant to ceftriaxone. The inclusion of the isolates that did not exhibit MDR or ceftriaxone resistance phenotypes could be either due to a loss of antibiotic resistance during freezing storage or due to a change in the breakpoints for certain antibiotics or the definition of MDR. The prevalence of antibiotic resistance phenotypes from freeze-stored samples was reported to be lower compared to the fresh samples [28], and since the *E. coli* isolates in this study had been stored for prolonged periods of time at −80 °C, some isolates may have lost resistance phenotypes. In fact, when the current MICs of the isolates were compared with their original MICs, four isolates were found to have decreased MICs that changed their resistance phenotypes (Table 2). An isolate (CRIS-Ec57) lost resistance to five antibiotics, including AugAmpFoxAxo and streptomycin, losing resistance towards β-lactams altogether. The pan-susceptible isolate (CRIS-Ec58) was originally resistant to multiple drugs, which were ciprofloxacin, nalidixic acid, gentamicin, streptomycin, and tetracycline. Another isolate (CRIS-Ec63) lost resistance to ciprofloxacin, nalidixic acid, and tetracycline, while still another isolate (CRIS-Ec82) lost resistance to tetracycline. CLSI occasionally revises the breakpoints for certain antibiotics as the prevalence of resistance towards the drugs increases and lowering of the breakpoints becomes essential to predict clinical outcomes [29]; however, lowering the breakpoints would lead to more isolates being considered resistant to the antibiotics, so the change in the breakpoints may not be the cause of the reduced number of MDR or ceftriaxone-resistant isolates in this study. In addition, while MDR was defined as resistance to three or more antibiotic classes for the current study, MDR could also be defined as resistance to more than one antibiotic class or resistance to multiple antibiotic drugs. The variation in the definition of MDR is a very likely reason for including the isolates that are not within our selection criteria.

### 3.2. Antibiotic Resistance Genes of E. coli Isolates

The genomes of all 100 *E. coli* isolates were sequenced. Table S1 depicts the genomic characteristics of the sequenced *E. coli* isolates, including the genome size, GC content, N50, and genome coverage of the isolates. The genome size of the isolates ranged from 4.9 Mb to 5.9 Mb with a GC content of 50.1 to 50.8 and coverage of 21 to 67.

**Figure 1:**
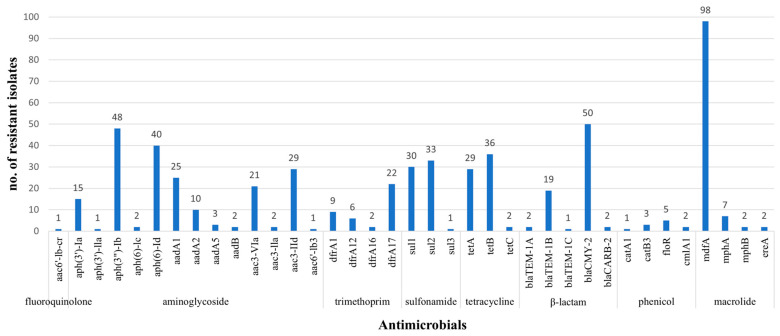
Antibiotic resistance genes detected in the genomes of 100 *E. coli* US poultry-associated isolates.

ResFinder analysis showed the presence of 37 antibiotic resistance genes among the poultry *E. coli* isolates (Figure 1). The most prevalent gene was *mdfA*, which was present in most of the isolates (*n* = 98). This resistance gene, known to confer resistance to multiple antibiotics, such as fluoroquinolones, erythromycin, chloramphenicol, and aminoglycosides [30], is reported to be prevalent in *E. coli* recovered from chicken samples from other geographical regions, including China, Pakistan, and Canada [31,32,33]. Of the 62 tetracycline-resistant isolates, 61 isolates carried either *tetA*, *tetB*, *tetC*, or combinations of two of the three tetracycline resistance genes. The most common gene was *tetB*, detected in 36 isolates, followed by *tetA* and *tetC*, which were detected in 29 and 1 isolates, respectively. Four isolates contained both *tetA* and *tetB*, while one isolate contained both *tetA* and *tetC*. None of the 38 tetracycline-susceptible isolates carried tetracycline resistance genes, except 1 isolate; this isolate carrying *tetC* exhibited an intermediate resistance phenotype against tetracycline.

All 49 isolates with the antibiotic resistance phenotype pattern of AugAmpFoxAxo harbored *bla*_CMY-2_, with 5 isolates carrying an additional *bla*_TEM-1B_ gene and 2 isolates carrying an additional *bla*_CARB-2_ (also known as *bla*_PSE-1_) gene. There were 17 isolates that exhibited resistance to only ampicillin, out of the four β-lactam antibiotics tested, and all carried a variant of *bla*_TEM_ (*bla*_TEM-1A_, *bla*_TEM-1B_, *bla*_TEM-1C_), with *bla*_TEM-1B_ being the most abundant variant gene (*n* = 14). Of the 34 isolates that did not exhibit resistance to any of the β-lactam drugs tested, 1 isolate was positive for the *bla*_CMY-2_ gene. The isolate was originally resistant to AugAmpFoxAxo but became susceptible to these β-lactam drugs upon retest, suggesting that there could be a reduced expression of the *bla*_CMY-2_ gene or a mutation in the gene that no longer confers resistance to β-lactam drugs. While *bla*_CMY-2_ was the most common β-lactamase gene in this study, identified in 50 isolates, the most common β-lactamase gene in poultry-associated *E. coli* differs by geographical locations [34,35,36]. Although 49 isolates were resistant to ceftriaxone, which is known to be an indicator for potential ESBL-producers, none of the isolates were positive for ESBL-encoding genes. This demonstrates that ceftriaxone, a third-generation cephalosporin, is not a good indicator to screen for ESBL producers in the US where *bla*_CMY-2_ is prevalent.

Three sulfisoxazole resistance genes, *sul1*, *sul2*, and *sul3*, were detected in 30, 33, and 1 of the 42 sulfisoxazole-resistant isolates, respectively, with 22 isolates containing both *sul1* and *sul2* genes. While all 42 isolates with the sulfisoxazole resistance phenotype carried one or two sulfisoxazole resistance genes, none of the isolates with the sulfisoxazole-susceptible phenotype carried the resistance genes. In the 19 trimethoprim/sulfamethoxazole-resistant isolates, either *dfrA1* (*n* = 9), *dfrA12* (*n* = 6), or *dfrA17* (*n* = 5) was detected, with 1 isolate positive for both *dfrA1* and *dfrA17* genes. While all resistant isolates were positive for one or more genes that might be responsible for the trimethoprim/sulfamethoxazole resistance phenotype, 23.5% (19/81) of the trimethoprim/sulfamethoxazole-susceptible isolates were also positive for the resistance genes; 2 isolates carried *dfrA16,* while 17 isolates carried *dfrA17*.

Streptomycin and gentamicin were the only aminoglycoside antibiotics tested in this study, while 13 different aminoglycoside resistance genes were found among the 100 sequenced isolates. Although there are no CLSI-approved breakpoints for streptomycin, 54 isolates exhibited increased MICs (≥64 μg/mL) for the drug. *aph(3″)-Ib* (*n* = 48), *aph(6)-Id* (*n* = 40), *aac(3)-IId* (*n* = 29), *aadA1* (*n* = 25), and *aac(3)-VIa* (*n* = 21) were the most prevalent aminoglycoside resistance genes found in both the resistant and susceptible isolates, suggesting that these antibiotic resistance gene-positive isolates might be resistant to aminoglycoside antibiotics that were not included in this study. On the other hand, there were seven isolates that were negative for the aminoglycoside resistance genes but were still resistant to streptomycin, with or without resistance to gentamicin, showing that the aminoglycoside resistance phenotypes and in silico search of resistance genes do not align. An improvement in the antibiotic resistance database may help reduce the discrepancy between the resistance phenotype and the genotype predicted by the database. *aac(6′)-Ib-cr* encodes an aminoglycoside acetyltransferase that also confers reduced susceptibility to ciprofloxacin and only 1 out of 42 ciprofloxacin-resistant isolates carried this gene [37].

The chromosomal mutations within quinolone resistance-determining regions of DNA gyrase and topoisomerase IV were likely responsible for the (fluoro)quinolone resistance observed in the *E. coli* isolates as all the resistant isolates had at least one mutation in these regions. All 46 nalidixic acid-resistant isolates, out of which 42 isolates exhibited resistance to ciprofloxacin as well, showed mutations in *gyrA*, *parC*, and/or *parE* (Table 3), including the 1 ciprofloxacin-resistant isolate harboring *aac(6′)-Ib-cr*. Four isolates that were resistant to only nalidixic acid had single mutations in *gyrA* and no mutations in *parC*. In contrast, those isolates that were resistant to both nalidixic acid and ciprofloxacin showed double mutations in *gyrA* and either single or double mutations in *parC*, along with single mutations in *parE* in most of the isolates. An S83L mutation in the *gyrA* gene was the most common mutation (*n* = 44), while other mutations observed in *gyrA* were D87N (*n* = 39), D87Y (*n* = 4), and A84P (*n* = 1) substitutions. All the ciprofloxacin-resistant isolates had mutations in *parC* at either position 80 [S80I (*n* = 41) or S80R (*n* = 1)] and/or position 84 [E84G (*n* = 24), E84A (*n* = 1), or E84K (*n* = 1)] with an additional amino acid substitution, A108T, present in an isolate. A mutation I255T in the *parE* gene was observed in 25 ciprofloxacin-resistant isolates, 24 of which also showed a *parC* E83G mutation, indicating that there may be an association between these two mutations. Another amino acid substitution, S458A, was observed in ciprofloxacin-resistant isolates (*n* = 6).

Seven *E. coli* isolates were positive for *mphA*, and they all exhibited increased MICs for azithromycin. On the other hand, four isolates positive for other macrolide genes, either *mphB* or *ereA*, did not exhibit increased MICs for azithromycin. These isolates may be resistant to other macrolides that were not included in the current study as azithromycin was the only macrolide drug tested. The detection of *floR*, *cmlA1*, *catA1*, and *catB3* was observed in seven chloramphenicol-resistant isolates. Five isolates harbored *floR*, and two isolates carried *cmlA1*, with one isolate containing both the resistance genes, while one chloramphenicol-resistant isolate co-harbored *catA1* and *catB3*.

Antibiotic resistance and the resistance mechanisms in *E. coli* are similar to those found in *Salmonella* [38]; thus, the antibiotic resistance genes identified in our poultry-associated *E. coli* isolates can be transferred to *Salmonella*, pathogenic bacteria which are also commonly found in poultry sources. Antibiotic-resistant *E. coli* could serve as a reservoir and donor of resistance to *Salmonella*, and resistant *Salmonella* can spread through the food chain and cause infections in humans. Infections caused by antibiotic-resistant *Salmonella* are a public health concern as they can be more severe than infections caused by susceptible bacteria and harder to treat due to fewer treatment options [8,39].

### 3.3. Mobile Genetic Elements (Plasmids and Phages) of E. coli Isolates

**Figure 2:**
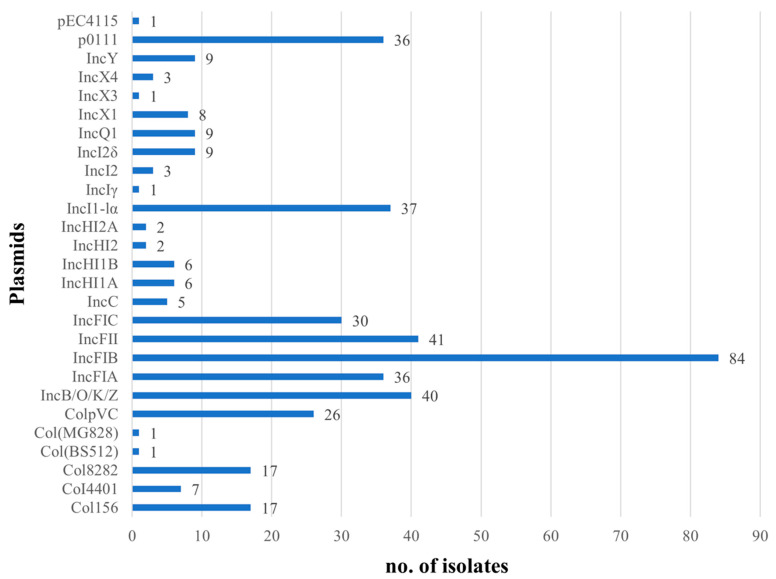
Plasmid replicons identified in the genomes of 100 US poultry-associated *E. coli* isolates.

All sequenced *E. coli* isolates included in this study harbored at least one plasmid replicon. A whole-genome sequence analysis identified 27 different plasmid replicons among the isolates, with IncFIB being the most common replicon, which was detected in 84% of the isolates (Figure 2). With the exception of an isolate with a single plasmid replicon, the remaining 99 isolates carried multiple replicons. Most isolates (74%) contained three to five plasmid replicons, with up to seven replicons per isolate. The majority of isolates (92%) harbored at least one IncF plasmid (IncFIA, IncFIB, IncFII, IncFIC), out of which two isolates carried all four IncF plasmids. This was in agreement with previous studies that identified a high prevalence rate of IncF replicon types among *E. coli* isolates from poultry rinsate samples [40,41,42]. In addition to IncF plasmids, IncB/O/K/Z, IncI1-lα, and p0111 were frequently identified as they were each harbored by approximately 40% of the isolates.

**Figure 3:**
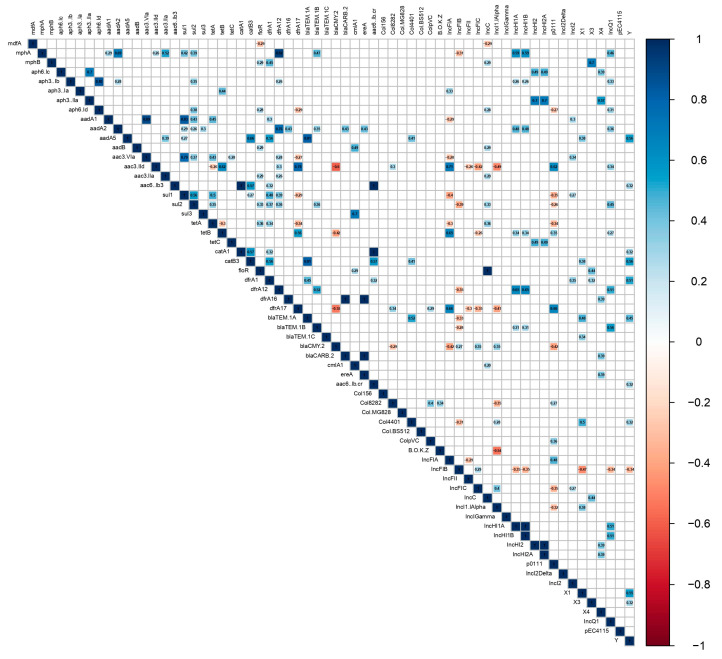
Correlation analysis between resistance genes and plasmid replicons among 100 US poultry-associated *E. coli* isolates. Blue and red colors of boxes indicate positive and negative correlation, respectively. The strength of the colors corresponds to the numerical value of the correlation coefficient (r). Numbers within boxes denote correlation coefficient (r).

The relationship between antibiotic resistance genes and plasmid replicon types among the examined *E. coli* isolates was determined using a correlation analysis (Figure 3). The results showed the presence of significant strong positive correlations (r > 0.6) among genes conferring resistance to different antibiotic classes as well as between resistance genes and plasmid replicon types among the examined isolates. An r value of 1 was observed among *bla*_CARB-2_, *dfrA16*, and *ereA*. Although this gene combination was detected only in two isolates, a contig carrying *ereA*, *bla*_CARB-2_, *dfrA16*, and *aadA2* was also detected in *Salmonella* serovar Tennessee, which was identified as an *E. coli* integron *int*l1 (KX57988) [43]. The two *E. coli* isolates from the present study also harbored *aadA2*, but the r values of *aadA2* with the other three genes were 0.48. Similarly, an r value of 1 was observed among *aac(6′)-Ib-cr*, *aac(6′)-Ib3*, and *catA1*, although this antibiotic resistance gene combination was detected in one isolate. A strong positive correlation (r = 1) was also detected between the IncC plasmid and *floR* in five isolates, indicating that this chloramphenicol resistance gene is mainly located on the IncC plasmid in *E. coli* from poultry rinsate samples.

*E. coli* isolated from poultry rinsate samples contained a wide array of phages. Most isolates (95%) harbored at least one intact phage, with the majority of the isolates (72%) carrying two to four phages. While only eight isolates had a single phage identified, as many as seven intact phages were identified in an isolate. Phages varied in size, ranging from 5.8 kb to 111 kb in length. Enterobacteria phage mEp460 (PHAGE_Entero_mEp460_NC_019716) was the most prevalent phage, found in 30 isolates, followed by *Escherichia* phage P88 (PHAGE_Entero_P88_NC_026014) and Stx2-converting phage 1717 (PHAGE_Stx2_c_1717_NC_011357), which were both identified in 23 isolates. Stx2-converting phages carry the *stx2* gene, which encodes the Shiga toxin 2, and transfer this virulence factor to other bacteria through horizontal gene transfer, potentially leading to the development of Stx2-producing Shiga toxin-producing *E. coli* (STEC) [44]. In addition to *Escherichia* phages, phages from *Burkholderia*, *Flavobacterium*, *Haemophilus*, *Pectobacterium*, *Pseudomonas*, *Ralstonia*, *Salmonella*, *Shigella*, *Vibrio*, and *Yersinia* were also identified. Prophages can be incorporated into a bacterial genome, thus introducing genetic elements and increasing the fitness of the host; however, these genes can also be mobilized and transferred to a new host. The prophages detected among the *E. coli* isolates may facilitate the acquisition and transfer of the resistance genes they carry.

### 3.4. Heatmap Clustering and Phylogenetic Analysis of the Examined E. coli Isolates

**Figure 4:**
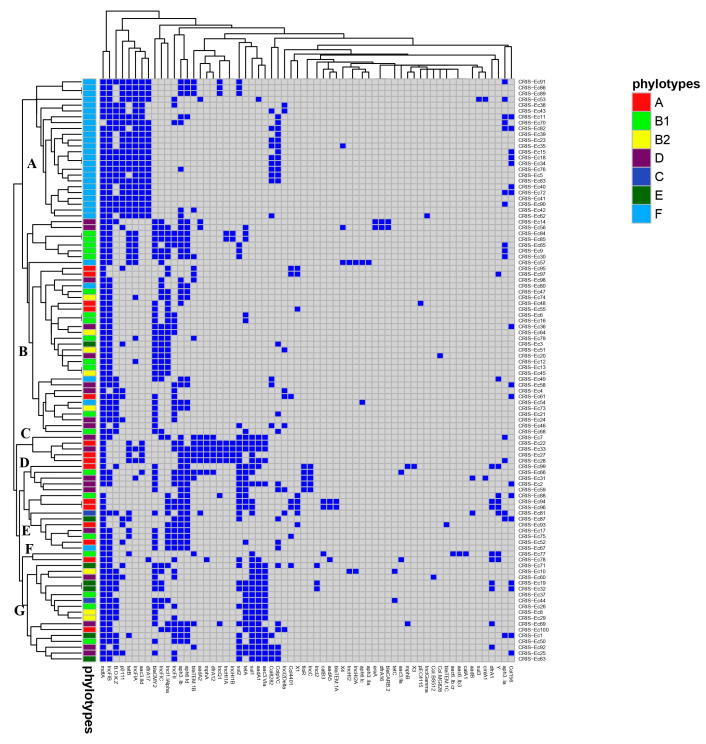
A heatmap supported by a dendrogram demonstrating the distribution of 100 US poultry-associated *E. coli* according to their antimicrobial resistance genes and plasmid replicons. Dark blue represents the presence of resistance genes and plasmid replicon types, and gray represents the absence of genes and plasmid replicons. Seven clusters (A–G) are indicated in the figure.

The overall distribution of antimicrobial resistance genes and plasmid replicon types among the examined *E. coli* isolates with different phylotypes was determined using a heatmap with hierarchical clustering. The generated heatmap revealed the clustering of the 100 *E. coli* isolates into seven major clusters (A–G) based on their resistance genotype and plasmid replicon profiles (Figure 4). Cluster B was the largest cluster, composed of 37 isolates. Isolates grouped in each cluster were assigned to different phylotypes, except cluster A, where all isolates in this cluster were assigned to the same phylotype, F. The similarity in antibiotic resistance gene and plasmid replicon carriage indicates a lack of genetic diversity within phylotype F compared to other phylotypes. Seven pairs of isolates were identical: two pairs in cluster A (CRIS-Ec86 and CRIS-Ec89, and CRIS-Ec18 and CRIS-Ec34), three pairs in cluster B (CRIS-Ec84 and CRIS-Ec85, CRIS-Ec6 and CRIS-Ec16, and CRIS-Ec13 and CRIS-Ec45), and one pair each in cluster E (CRIS-Ec94 and CRIS-Ec96) and G (CRIS-Ec19 and CRIS-Ec32). Isolates in identical pairs had identical profiles of antibiotic resistance genes and plasmid replicons and were assigned to identical phylotypes, except isolates CRIS-Ec13 and CRIS-Ec45 that belonged to different phylotypes.

**Figure 5:**
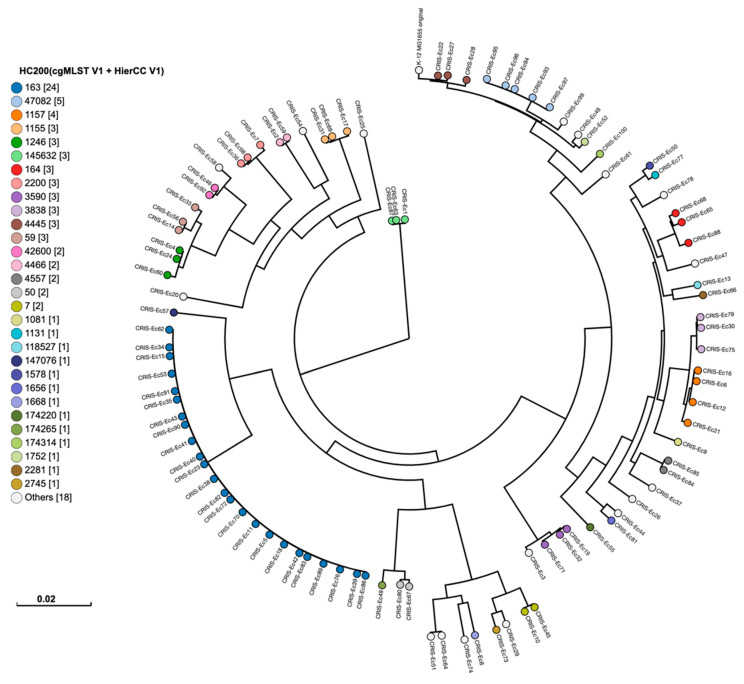
Single-nucleotide polymorphisms (SNPs) and hierarchical clustering of cgMLST (HierCC) of 100 US poultry-associated *E. coli* isolates. The legend shows the cgMLST HC200 that indicates allelic differences no more than 200 of 2513 core genomic alleles among isolates. The numbers in brackets indicate the number of isolates assigned to each HC200 pattern.

To determine the epidemiological relationship among the examined *E. coli* isolates, phylogenetic analysis based on SNPs and HierCC of cgMLST was performed (Figure 5). At the hierarchical cluster (HC) level HC200 that shows ≤200 differences in core genomic alleles, the examined isolates were classified into 30 patterns. Isolates with the same pattern contained similar core genomic alleles and differed only by 200 alleles of the *E. coli*’s core genomic loci (*n* = 2413). HC200|163 was the predominant pattern that encompassed 24 isolates, followed by HC200|47082 in 5 isolates and HC200|1157 in 4 isolates. The 24 isolates that belonged to the same HC200|163 pattern presented similar antibiotic resistance gene profiles; they all carried *aac3-IId* (24/24), while only 5 isolates carried the gene out of the remaining 76 isolates that belonged to other patterns. Similarly, 20 out of 24 isolates (83%) carried *tetB*, whereas 16 out of 76 isolates (21%) with other patterns carried the gene. The presence of *dfrA17* among the HC200|163 isolates was 87.5% (21/24), while the presence of the gene among the non-HC200|163 isolates was 1.3% (1/76). In addition, all isolates belonging to this pattern carried IncFIA, IncFIB and p0111. The prevalence of the HC200|163 pattern among the highly diverse *E. coli* isolates indicates the circulation of this clone in US chicken over multiple years.

## 4. Conclusions

In this study, the genomes of 100 antibiotic-resistant *E. coli* isolates associated with poultry rinsate samples of chicken meat intended for human consumption were sequenced. The genomic data were used to study antibiotic resistance genes that can potentially be transferred to other bacteria as well as mobile genetic elements, which can facilitate the transfer. Diverse genes conferring resistance to different antibiotic families and mobile genetic elements were identified; 37 AR genes and 27 plasmid replicons were predicted among the 100 sequenced isolates, and 50 different phages were predicted among 95 isolates. *E. coli* isolates in the present study harbored multiple plasmids belonging to various replicon types as well as multiple phages that were originally from different host bacteria.

Generating and analyzing genomic data on resistant *E. coli* can facilitate understanding of the prevalence and diversity of antibiotic resistance genes and mobile genetic elements in *E. coli* from poultry rinsate samples as well as the potential for the transmission of resistance genes via the food chain. Genomic data can also be useful in finding gene targets useful for providing solutions to limit the spread of antibiotic resistance. *E. coli* isolates analyzed in the current study are from our previous collection of isolates recovered between 2001 and 2012 before more strict regulations were enforced on antibiotic use on food animals, including the ban of extra-label use of cephalosporins in food-producing animals in 2012 and the ban of antibiotic use as growth promoters in livestock production in the US in 2017 [45,46]. As future research, comparing the sequences of our isolates with the sequences of more recent *E. coli* isolates associated with poultry will help us understand the trends of resistance genes and the genetic elements responsible for their transmission in the poultry settings and if the regulations and guidelines have helped mitigate the problem of antibiotic resistance in food animals in the US.

## Figures and Tables

**Table 1:** Antibiotic resistance phenotype patterns in 100 US poultry-associated *E. coli* isolates.

Antibiotic Resistance Phenotype Pattern *	No. of Resistance (Drug)	No. of Resistance (Class)	No. of Isolates
Pan-susceptible	0	0	1
Nal	1	1	1
Str	1	1	1
Tet	1	1	1
AmpCipNal	3	2	1
CipGenNal	3	2	4
CipNalStr	3	2	2
GenStrFis	3	2	1
AugAmpFoxAxo	4	1	13
AmpCipNalStr	4	3	3
CipGenNalTet	4	3	12
GenStrFisTet	4	3	1
AugAmpFoxAxoNal	5	2	1
AugAmpFoxAxoStr	5	2	4
AugAmpFoxAxoFis	5	2	1
AugAmpFoxAxoTet	5	2	6
CipGenNalStrTet	5	3	3
CipGenNalFisTet	5	4	1
CipNalStrFisTet	5	4	2
GenNalStrFisTet	5	4	2
AugAmpFoxAxoStrTet	6	3	4
AmpCipGenNalStrTet	6	4	1
AziCipGenNalFisSxt	6	4	1
AugAmpFoxAxoCipNalFis	7	3	1
AugAmpFoxAxoGenStrFis	7	4	3
AugAmpFoxAxoStrFisTet	7	4	3
AmpAziGenStrFisTetSxt	7	5	5
AmpChlCipNalFisTetSxt	7	5	1
AmpCipNalStrFisTetSxt	7	5	2
ChlCipGenNalFisTetSxt	7	5	1
AugAmpFoxAxoChlStrFisTet	8	5	1
AugAmpFoxAxoGenStrFisTet	8	4	4
AugAmpFoxAxoStrFisTetSxt	8	4	1
AmpCipGenNalStrFisTetSxt	8	5	4
AugAmpFoxAxoChlGenStrFisTet	9	5	2
AugAmpFoxAxoGenStrFisTetSxt	9	4	2
AugAmpFoxAxoCipGenNalStrFisTet	10	5	1
AugAmpFoxAxoChlCipNalStrFisTetSxt	11	6	1
AugAmpAziFoxAxoChlCipGenNalStrFisTetSxt	13	7	1

* Abbreviations: amoxicillin/clavulanic acid (Aug), ampicillin (Amp), azithromycin (Azi), cefoxitin (Fox), ceftriaxone (Axo), chloramphenicol (Chl), ciprofloxacin (Cip), gentamicin (Gen), nalidixic acid (Nal), streptomycin (Str), sulfisoxazole (Fis), tetracycline (Tet), trimethoprim/sulfamethoxazole (Sxt).

**Table 2:** The original MICs and the retested MICs of the four *E. coli* isolates against 13 antibiotic drugs.

Antibiotics	Minimum Inhibitory Concentrations (mg/mL) *							
	CRIS-Ec57		CRIS-Ec58		CRIS-Ec63		CRIS-Ec82	
	Original	Retested	Original	Retested	Original	Retested	Original	Retested
amoxicillin/clavulanic acid	**32**	4	4	4	4	4	4	4
ampicillin	**>32**	4	4	4	2	2	2	4
azithromycin	N/A	4	N/A	4	N/A	2	N/A	2
cefoxitin	**>32**	2	8	4	4	4	8	4
ceftriaxone	**16**	≤0.25	≤0.25	≤0.25	≤0.25	≤0.25	≤0.25	≤0.25
chloramphenicol	8	8	4	4	8	4	8	4
ciprofloxacin	≤0.015	≤0.015	**>4**	≤0.015	**>4**	≤0.015	**>4**	**>4**
gentamicin	0.5	0.5	**>16**	≤0.25	**>16**	**>16**	**>16**	**>16**
nalidixic acid	2	4	**>32**	1	**>32**	2	**>32**	**>32**
streptomycin	**64**	8	**>64**	32	**>64**	**64**	≤32	8
sulfisoxazole	≤16	32	≤16	32	**>256**	**>256**	32	≤16
tetracycline	**>32**	**>32**	**>32**	≤4	**32**	≤4	**>32**	≤4
trimethoprim/sulfamethoxazole	≤0.12	≤0.12	1	≤0.12	1	1	0.5	0.5

* Bold letters indicate the resistance phenotypes.

**Table 3:** Amino acid substitutions in mutations of the quinolone resistance-determining regions in poultry-associated *E. coli* isolates with the quinolone resistance phenotype.

Phenotypic Resistance To	Amino Acid Substitutions In								No. of Isolates
	*gyrA*			*parC*			*parE*		
nalidixic acid	S83L	-	-	-	-	-	I355T	-	1
	-	-	D87Y	-	-	-	-	-	2
	S83L	-	-	-	-	-	-	-	1
ciprofloxacin + nalidixic acid	S83L	-	D87N	S80I	E84G	-	I355T	-	24
	S83L	-	D87N	S80I	E84A	-	-	-	1
	S83L	-	D87N	S80I	-	A108T	-	-	1
	S83L	-	D87N	S80I	-	-	I355T	-	1
	S83L	-	D87N	S80I	-	-	-	S458A	6
	S83L	-	D87N	S80I	-	-	-	-	5
	S83L	-	D87N	S80R	-	-	-	-	1
	S83L	-	D87Y	S80I	-	-	-	-	2
	S83L	A84P	-	-	E84K	-	-	-	1

## Data Availability

Data underlying this article are available in the BioProject database of the National Center for Biotechnology Information at https://www.ncbi.nlm.nih.gov/bioproject/ (accessed on 18 May 2025) and can be accessed with PRJNA828150 and PRJNA1112887.

## Supplementary Materials

The following supporting information can be downloaded at: https://www.mdpi.com/article/10.3390/pathogens14080726/s1, Table S1: Masterfile for the *E. coli* isolates from poultry.
